# Local Treatment in Diphtheria

**Published:** 1877-03

**Authors:** A. W. Hagenbuch

**Affiliations:** Assistant Physician at Cook County Insane Asylum and Poor House


### LOCAL TREATMENT IN DIPHTHERIA, WITH CASES.

By A. W. HAGENBUCH, M.D.,
Assistant Physician at Cook County Insane Asylum and Poor House.

In giving a sketch of an epidemic of diphtheria which came under my care at the Cook county pauper institution, I will strive to notice the main features of the epidemic as they presented themselves.

The total number of patients treated was thirty-three; this number including only cases with well marked pseudo-membrane, all cases of simple pharyngitis being excluded. Of this number six were males, twenty-seven females. The greater number of females attacked is explained by the fact that all boys over five years of age are removed to a separate apartment, while the girls are allowed to remain with their mothers.

The first case was brought to my notice December 9th, 1876. The last, January 13th, 1877. The rate of increase was more marked at the beginning than towards the close of the epidemic.

The average duration, counting from first appearance to the complete disappearance of the pseudo-membrane, was about ten days. In one case I made daily local applications for seventeen successive days.

The tonsils were the primary seat of the pseudo-membrane in ten cases; the soft palate was involved in thirteen cases; the larynx in two cases, one of which proved fatal. The entire nasal cavity was involved in one case, a thin, ichorous discharge resulting, and upon several occasions a severe hæmorrhage followed the act of coughing or sneezing. The post-pharyngeal wall alone was involved in a single case. In the remaining six cases the hard palate, cheeks, etc., were the seat of the pseudo-membrane.

The only fatal case was that of Willie T., aged twelve months. He was attacked suddenly January 3rd, and, although strong and healthy up to this date, was rapidly prostrated. Local applications could not be employed thoroughly. The larynx was involved by extension of the inflammation on the third day; the characteristic croupous cough was marked, and the patient died on the fifth day, of asphyxia.

Most of the causes generally supposed to operate in the production of diphtheria have been present to an unusual extent; improper hygienic surroundings, crowded apartments, and the diet, although better than the pauper element of large cities can afford, does not contain in sufficient quantities all the proximate principles necessary for the perfect reproduction of the rapidly changing tissues of the growing child; milk, especially, is very scarce during the winter months.

That the disease is but slightly contagious may be proven by the fact that of the one hundred and sixty children inmates, freely intermingling in the school-room and different houses, only those in house C were attacked. The boys' room, about two hundred feet distant, with eighty inmates, did not furnish a single case. I believe the diphtheria poison (whatever that may be) to be slightly communicable by contagion. The fact that I was the only sufferer from diphtheria in the Insane Asylum where I reside, proves to my mind that the close contact with patients, while making the local applications, accounts for the attack in my case. The possibility of having accidentally received some of the secretions into my mouth, and consequent inoculation, must not be lost sight of.

The experiments of Peters*, in which, by the application of a diphtheritic secretion, he was able to change the nature of any simple inflammation, so as to form false membranes upon ulcers, erosions, or any denuded surface, prove, beyond dispute, the inoculability of the diphtheritic secretions.

Paralysis of the soft palate was present in thirty per cent. of all the cases, and albumen in the urine was found in two out of five specimens examined. The paralysis of the soft palate prevented often the ingestion of liquid food, and lessened the facility with which local applications were made to the post-pharyngeal wall. Several cases, towards the close of the

* See Edinburg Med. Jour., 1860.

epidemic, suffered with severe diarrhœa, but improved with mild astringent preparations and good diet.

I ascribe the low rate of mortality, to some extent, at least, to the early and thorough local treatment employed.

Twenty cases, in which a vigorous local treatment was employed, made a more rapid and perfect recovery than cases in which the local treatment consisted only of weak astringent gargles, too early discontinued. In several cases I was obliged to return to the local applications to relieve suffering and imminent danger to life from accumulated secretions and extensive false membranes obstructing the pharyngeal space.

The local applications mostly employed consisted of equal parts of tincture of the chloride of iron and dilute nitric acid. First rinsing the mouth, and carefully removing the secretions and detached membranes with a dry swab, I applied the above preparation with a soft brush. The parts, upon inspection, now presented a dryer and more healthy appearance, the astringent application causing the contraction of the tissues, and giving to the enlarged tonsils a corrugated appearance. In three cases only was a stronger preparation than the above employed, including my own case, in which the nitric acid C. P. was carefully applied to the false membrane with an ivory probe; and although the membranes reappeared in other locations, a single slight application would completely remove the newly formed membrane, leaving in its place a healthy appearing granulated surface, which healed kindly without any further trouble.

To recapitulate: The marked features of the epidemic were the limitation of the affection to one house, showing its very slightly contagious nature; the rapid invasion and unfavorable progression if not interfered with; the low rate of mortality; the anæmia which follows; and above all, the unmistakable benefit derived in nearly every case in which vigorous local treatment was employed. In all cases, no matter what general treatment was employed, if the local applications were discontinued for a single day, the case was invariably worse; especially the cough and difficulty of breathing were aggravated.

